# Histopathological Scoring of Renal Injury in an Experimental Model of Acute Pancreatitis Caused by *Karwinskia humboldtiana* Fruit: Exploring Its Potential Utility in the Treatment of Pancreatic Cancer

**DOI:** 10.3390/ph18111652

**Published:** 2025-11-01

**Authors:** Nallely Esparza-Rodríguez, Nestor Jaime Solís-Flores, Gabriela Guadalupe Medellín-Zapata, Juan Carlos Segoviano-Ramírez, Katya Carcaño-Díaz, Marta Ortega-Martínez, Gilberto Jaramillo-Rangel, Jaime García-Juárez

**Affiliations:** 1 Instituto Mexicano del Seguro Social (IMSS), Hospital General de Zona No. 50, Servicio de Medicina Interna, San Luis 78397, San Luis Potosí, Mexico; esparza.ner@gmail.com; 2 Departamento de Tumores Mamarios, Instituto Nacional de Cancerología (INCan), Secretaría de Salud, Mexico City 14080, Mexico; njsf87660@gmail.com; 3 Servicio de Ginecología y Obstetricia, Instituto Mexicano del Seguro Social (IMSS), Hospital General de Subzona y Medicina Familiar No. 2, Cozumel 77664, Quintana Roo, Mexico; gabriela.medellin@hotmail.com; 4 Independent Researcher, García 66036, Nuevo León, Mexico; drjuancarlos.segoviano@live.com.mx; 5 Facultad de Medicina, Universidad Autónoma de Yucatán, Mérida 97000, Yucatán, Mexico; k.carcano@virtual.uady.mx; 6 Departamento de Patología, Facultad de Medicina, Universidad Autónoma de Nuevo León (UANL), Monterrey 64460, Nuevo León, Mexico; marta.ortegamrt@uanl.edu.mx (M.O.-M.); gilberto.jaramillorn@uanl.edu.mx (G.J.-R.); 7 Departamento de Histología, Facultad de Medicina, Universidad Autónoma de Nuevo León (UANL), Monterrey 64460, Nuevo León, Mexico

**Keywords:** *Karwinskia humboldtiana*, pancreatic cancer, kidney injury, histopathological scoring

## Abstract

**Background**: *Karwinskia humboldtiana* (*Kh*) is a toxic plant that produces a fruit that, when ingested in large quantities, causes damage to the lungs, liver, kidneys, pancreas, and duodenum. Pancreatic damage was measured using a semiquantitative score, revealing that it is progressive and characterized by selective apoptosis and necrosis of the exocrine portion. However, renal injury has not been evaluated using injury scales and has only been reported descriptively. Pancreatic adenocarcinoma is one of the top five causes of cancer death in the United States, and options for its treatment are limited. One of the toxins extracted from the fruit (T-514) has been tested as a possible antineoplastic agent in various cancer cell lines. In order to preliminarily assess its possible usefulness against pancreatic cancer, we decided to re-evaluate kidney damage using a semi-quantitative scale, hoping to obtain a lesser injury intensity than that reported in the pancreas. **Methods**: We analyzed kidney samples from the same model of acute pancreatitis by *Kh* by semiquantitative scoring to compare the results with those previously reported in the pancreas, and performed a TUNEL assay to analyze cell death in the kidney. **Results**: The renal injury that occurs in this model of *Kh* poisoning consists mainly of hydropic degeneration and loss of microvilli in proximal tubules. According to the scale used in this work, the following percentages of kidney injury were obtained: 8.26 ± 0.93% for the control group, 9.65 ± 1.60%, 11.04 ± 1.36%, 12.78 ± 2.46%, 14.03 ± 1.83% and 15.76 ± 3.73% for the groups 24 h, 48 h, 72 h, 96 h and 120 h after *Kh* administration. In contrast, the following were reported for the pancreas: 0.28 ± 0.83% for the control group, 14.81 ± 7.64%, 35.63 ± 12.05%, 67.13 ± 5.27%, 85.28 ± 13.14% and 87.13 ± 11.17% for the groups 24 h, 48 h, 72 h, 96 h and 120 h after *Kh* administration. These results indicate that pancreatic injury is 71.37% more intense than renal injury at 120 h after *Kh*. No evidence of nuclear chromatin fragmentation was found in the kidney. **Conclusions**: The renal damage in this model of acute pancreatitis is of lower intensity than the pancreatic damage, suggesting that *Kh* and its toxins may be useful in the treatment of pancreatic cancer and their study in an in vitro or in vivo cancer model is justified.

## 1. Introduction

*Karwinskia humboldtiana* (*Kh*) is a plant of the *Rhamnaceae* family found in Mexico, the southern United States, Central America, and the Caribbean; it produces a fruit with a highly toxic seed [1]. Accidental ingestion of the whole fruit in small quantities causes a progressive and ascending symmetrical paralysis similar to Guillain–Barré syndrome; in this case, the clinical features could be reversed within a few weeks [2,3]. The histopathological lesion described in paralysis is segmental demyelination of peripheral nerves [4], which has no specific treatment [5].

However, if the fruit is ingested copiously, it causes lesions in various organs that lead to the development of respiratory failure with fatal consequences with or without the development of paralysis [6]. These conditions have been studied in murine models, and it has been reported that during acute experimental poisoning, the administration of a single dose of 5 g/kg of *Kh* fruit produces inflammation of the proximal tubules and interstitial vascular congestion in the kidneys, centrilobular necrosis in the liver and generalized interstitial hemorrhage in the lungs [7,8].

In this experimental model, ripe *Kh* fruit causes a selective, progressive and intense injury in the exocrine portion of the pancreas characterized by apoptosis, necrosis and acute inflammation; a score of 0.06 ± 0.17 for the control group, 2.96 ± 1.53, 7.13 ± 2.42, 13.43 ± 1.05, 17.06 ± 2.63 and 17.43 ± 2.23 points for the groups 24 h, 48 h, 72 h, 96 h and 120 h after *Kh* administration were reported [9]. It has also been found that in this model of *Kh*-induced acute pancreatitis only the exocrine portion of the pancreas is affected and that the islets of Langerhans maintain the production of insulin and glucagon, thus preserving their morphological and functional integrity [10].

In the same way that the exocrine tissues of the pancreas are damaged, the submucosal Brunner glands present the formation of apoptotic bodies and necrosis; while in the duodenal wall there is edema of the intestinal villi, erosion of the intestinal epithelium with early loss of goblet cells followed by hyperplasia and vascular congestion in the lamina propria [11].

*Kh* seed contains various toxic anthraquinone-type compounds, which have been isolated and classified according to their molecular weight [12]. Of the isolated compounds, T-514 (Peroxisomycin A1 or PA1) has been evaluated as a potential antineoplastic agent due to its selective cytotoxic effect on hepatoma cancer cells, colon adenocarcinoma, lung adenocarcinoma, undifferentiated bronchogenic carcinoma, and small cell carcinoma [13,14].

Although selective toxicity to pancreatic cancer cells has not been evaluated, selective injury to pancreatic acini and islet of Langerhans integrity suggests that it may occur and that the isolated toxins may be considered for experimental use to study their potential utility as an antineoplastic agent in pancreatic cancer, which is one of the top five cancers causing death in the United States [15].

Because the reported damage to the pancreas was measured using a semiquantitative score and 17.43 ± 2.23 of 20 points were obtained at 120 h after administration of the *Kh* fruit [9], we considered it relevant to initiate a series of measurements in other organs, such as the kidney, in this model of acute pancreatitis, also using a semiquantitative score.

For this reason, in the present work we re-evaluated the renal injury caused by *Kh* fruit using a semi-quantitative scoring scale [16,17] in order to obtain a score that can be compared with those reported in pancreas [9] and to gather evidence to justify the study of its possible usefulness in the treatment of pancreatic cancer.

Furthermore, research is being conducted to improve the understanding of acute *Kh* fruit poisoning, to elucidate whether *Kh* causes direct damage affecting the various organs mentioned above or whether it has a selective toxic effect on the pancreas, which in turn leads to Systemic Inflammatory Response Syndrome (SIRS) as a result of the action of proinflammatory mediators [18,19,20,21], which subsequently causes Multiple Organ Dysfunction Syndrome (MODS) in which those organs are affected [22,23,24]. For this reason, it was decided to evaluate the effect of the ripe and whole *Kh* fruit in this work, because it poisons people and animals when consumed.

## 2. Results

### 2.1. Histopathological Analysis of the Kidney

**Figure 1:**
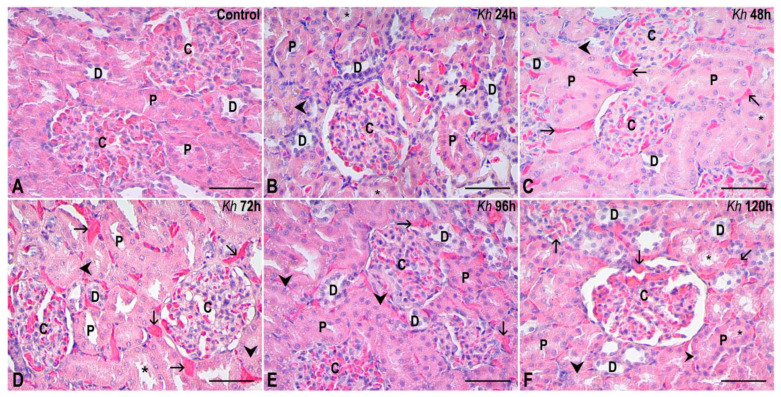
Representative sections of the renal cortex. (**A**) Kidney from the untreated control group. The renal corpuscles (C), proximal (P) and distal (D) tubules show a normal appearance. (**B**–**F**) Kidney samples from rats that received a single dose of *Kh* fruit obtained at 24 h, 48 h, 72 h, 96 h and 120 h after treatment, respectively. Renal corpuscles (C) are observed, maintaining an intact morphology that is similar to that observed in the control group without treatment. In contrast, the proximal tubules (P) and distal tubules (D) present some cells with hydropic degeneration (arrowheads) and some cells with microvilli that appear detached in some proximal tubules, revealing the acidophilic material that occupies the lumen in some of them (*); however, tubules with an appearance similar to the control are also present. Vascular congestion is observed in the interstitium (arrows). HE staining, observation with a 40× objective, scale bar: 50 µm.

**Figure 2:**
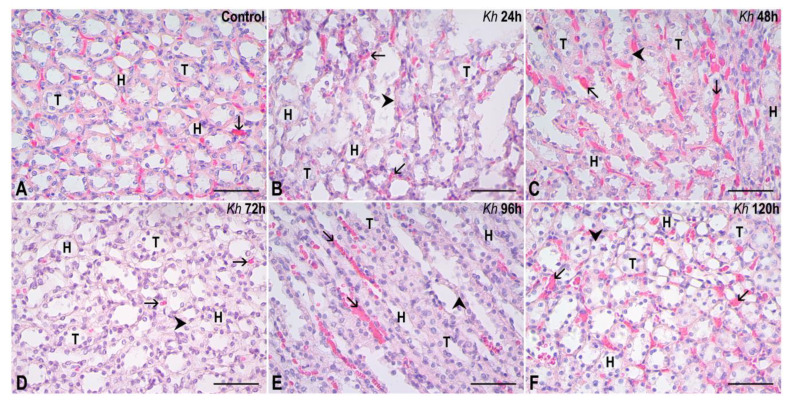
Representative sections of the renal medulla. (**A**) Kidney from the untreated control group. The collecting tubules (T) and the loops of Henle (H) show a normal appearance. (**B**–**F**) Kidney samples from rats that received a single dose of *Kh* fruit obtained at 24 h, 48 h, 72 h, 96 h and 120 h after treatment, respectively. It can be observed that the collecting tubules (T) and loops of Henle (H) have an epithelium with a morphologically intact appearance similar to that observed in the control group, except for some epithelial cells that present small vacuolizations indicating sites of hydropic degeneration (arrowheads). Vascular congestion is observed in the interstitium (arrows). HE staining, observation with a 40× objective, scale bar: 50 µm.

The lesions observed in histological sections of kidneys from rats treated with *Kh* were: microvilli detachment in the epithelium of the proximal tubules, diffuse hydropic degeneration, and some foci of necrosis. Vascular congestion, occasional erythrocyte extravasation, and some proteinaceous casts due to microvilli detachment were also observed (Figure 1). The lesions observed in the renal medulla were similar to those observed in the cortex and consisted mainly of vascular congestion and hydropic degeneration (Figure 2). No evidence of apoptotic bodies or inflammatory infiltrate was found in the glomeruli and renal interstitium of either the experimental or control groups.

### 2.2. Analysis by Semiquantitative Scoring of Kidney Damage

The mean scores obtained in the evaluation of each parameter, together with their standard deviations, are shown in Table 1. Values represented by 0.00 indicate that the evaluated parameter presented a preserved appearance similar to the control group.

**Figure 3:**
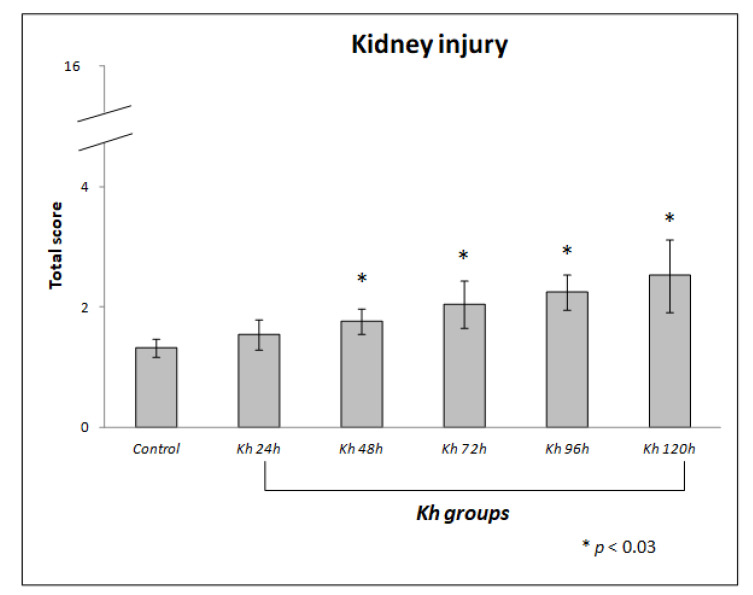
Score of renal histological damage in response to administration of ripe fruit of *Karwinskia humboldtiana.* Each bar represents the total score with its standard deviation. A one-way ANOVA and a Tamhane’s post hoc test, revealed significant difference between *Kh* and control groups, * (*p* < 0.03). There was no difference between control and 24 h after *Kh* group.

The mean scores obtained in the evaluation of kidney sections from untreated rats (control group) were 1.32 ± 0.15. While the means obtained in the kidney sections in rats treated with *Kh* fruit were: 1.54 ± 0.26 in the 24 h group, 1.77 ± 0.22 in the 48 h group, 2.04 ± 0.39 in the 72 h group, 2.24 ± 0.29 in the 96 h group and 2.52 ± 0.60 in the 120 h group (Figure 3).

We found a statistically significant difference from 48 h after *Kh* administration compared to the control group (*p* < 0.03).

### 2.3. Comparison of Renal and Pancreatic Injuries

The means and standard deviations were converted into percentages according to the maximum value of the kidney damage scale (16 points) and the following were obtained: 8.26 ± 0.93% for the untreated control group, 9.65 ± 1.60% for the 24 h after *Kh* group, 11.04 ± 1.36% for the 48 h after *Kh* group, 12.78 ± 2.46% for the 72 h after *Kh* group, 14.03 ± 1.83% for the 96 h after *Kh* group and 15.76 ± 3.73% for the 120 h after *Kh* group.

**Figure 4:**
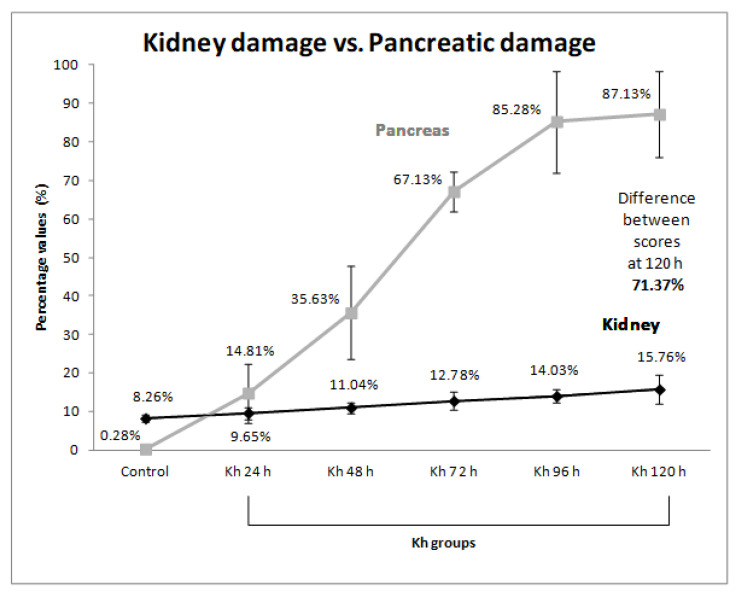
Comparison of the intensity of kidney damage versus pancreas damage. Each point in the graph shows the percentage score obtained for each group with its standard deviation. The gray line represents the previously reported pancreas damage score [9], and the dark line represents the kidney damage score. The greatest percentage difference (71.37%) between kidney and pancreas damage was observed in the group 120 h after *Kh* fruit administration.

Similarly, the percentages of each score obtained in the previous report on pancreatic damage [9] were obtained according to the maximum score (20 points). The percentages obtained for the pancreas were: 0.28 ± 0.83% (0.06 ± 0.17 points) for the untreated control group, 14.81 ± 7.64% (2.96 ± 1.53 points) for the 24 h after *Kh* group, 35.63 ± 12.05% (7.13 ± 2.42 points) for the 48 h after *Kh* group, 67.13 ± 5.27% (13.43 ± 1.05 points) for the 72 h after *Kh* group, 85.28 ± 13.14% (17.06 ± 2.63 points) for the 96 h after *Kh* group, and 87.13 ± 11.17% (17.43 ± 2.23 points) for the 120 h after *Kh* group. A progressive difference in the intensity of tissue injury was found between both organs depending on the observation time, observing the highest difference (71.37%) at 120 h after *Kh* (Table 2 and Figure 4).

### 2.4. The TUNEL Assay Was Negative in All Experimental Groups

**Figure 5:**
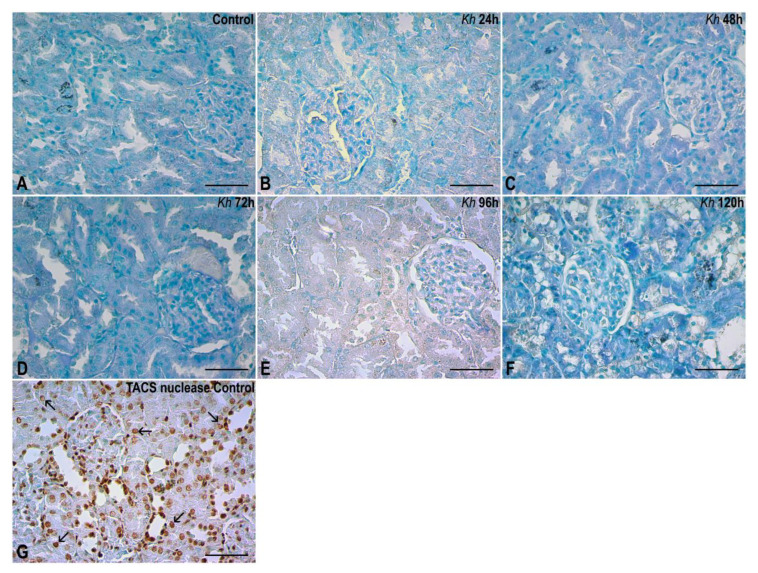
TUNEL assay. (**A**) Renal cortex of the untreated control group. No evidence of nuclear chromatin fragmentation was found in kidney. (**B**–**F**) Kidney cortex of samples from rats that received a single dose of *Kh* fruit obtained at 24 h, 48 h, 72 h, 96 h and 120 h after treatment, respectively. It is observed that the epithelial nuclei in renal corpuscles and tubules present a negative result similar to that observed in the control group. (**G**) Renal cortex of a sample from the untreated control group that was incubated with the TACS nuclease enzyme (internal control). Here the presence of positive labels to the TUNEL assay can be seen in both the renal corpuscles and tubules (arrows). Methyl green counterstaining, observation with a 40× objective, scale bar: 50 µm.

No positive signals for chromatin fragmentation were found in the nuclei of any of the experimental groups treated with *Kh* fruit or in the untreated control group. In the untreated rat kidney sample used as an internal control treated with TACS nuclease, a positive result was found, represented by brown staining in the nuclei of renal tubule and glomerulus cells, as expected according to the manufacturer’s instructions (Figure 5).

## 3. Discussion

It is known that the epithelia that form the renal tubules can be directly damaged by toxins, drugs or ischemia, which are the main causes of damage [25,26]. Cell death is evidenced by the demonstration of fragmented nuclear chromatin [27]; this has been reported in the kidney when a 2LD_50_ dose (28 mg/kg) of T-514 isolated from the species *Karwinskia parvifolia* is administered intraperitoneally [28].

In contrast, in this experimental model of acute pancreatitis, after administering a single oral dose of *Kh* fruit (5 g/kg), no apoptotic bodies or TUNEL-positive stains were observed in the kidney, even up to 120 h after *Kh* administration (Figure 5), whereas numerous apoptotic bodies and TUNEL-positive stains were reported in the exocrine portion of the pancreas of experimental groups at 72 h, and some stains at 96 and 120 h [9]. Meanwhile, the endocrine portion (islets of Langerhans) maintained its morphological and functional integrity [10].

In this study, we demonstrate that renal injury is not as severe as pancreatic injury when ripe *Kh* fruit is ingested, as evidenced by the scoring obtained from the quantified parameters (Table 1 and Figure 3).

Furthermore, the recorded histological evidence also demonstrates that, although samples from the experimental groups treated with *Kh* exhibited alterations such as hydropic degeneration and loss or detachment of apical microvilli in proximal tubules, tubules were also observed that maintained a histological appearance similar to the untreated control group (Figure 1 and Figure 2).

In contrast, in other experimental models of renal injury, such as that induced by a single oral dose of 200 mg/kg of chloroform, significant histopathological damage is observed, characterized by dilation of the renal tubules and the presence of casts from the first 24 h after administration [29].

Other phenomena that cause injury to the renal epithelium are usually nonspecific and caused by a variety of agents; in these cases, necrosis may occur due to the interruption of ATP production, mitochondrial injury, free radical formation, peroxidation, or altered cell signaling [30].

The T-514 and T-544 toxins isolated from *Kh* have been reported to reduce mitochondrial metabolism in hepatocytes in vitro [31] and the *Kh* fruit to reduce ATP concentration in vivo [32]; however, this has not yet been correlated with duodenal or pancreatic injury.

In the *Kh*-induced neuropathy model, thickening of the renal glomerular capsule and basement membrane has been reported [33], but these alterations were not observed in the present study.

Some chemotherapeutic agents, such as 5-fluorouracil (5-FU), cause thickening of basement membranes accompanied by loss of apical microvilli and basal folds [25,34], which partially coincides with what we observed in proximal tubules, because we only observed microvilli loss in this study.

The hydropic degeneration observed in tubular epithelia is consistent with reports of renal injury caused by the use of chemotherapeutic agents such as hydroxychloroquine [35], but the marked tubular dilation, casts, and edema reported with the use of these agents were also not observed in this study.

The observed cylinders were barely perceptible in the samples from the groups treated with *Kh*, but showed statistical significance only in the *Kh* 120 h group with respect to the control (*p* < 0.03). They could be formed by uromodulin, a protein with a protective function against infections and harmful agents [36], but we consider that it is necessary to explore longer experimental times in the future.

Although the kidneys are very sensitive to the adverse effects of chemicals and drugs [37], after administration of *Kh* fruit in this experimental model of acute pancreatitis, we observed that the renal injury was less severe than that previously reported in the pancreas (Table 2 and Figure 4).

In severe acute pancreatitis, systemic inflammatory response syndrome (SIRS) develops as a consequence of the action of proinflammatory mediators, usually within the first two weeks. This can lead to pulmonary, cardiovascular, hepatic, and renal failure [18,19,20,21].

Severe acute pancreatitis causes a 37.5–44.0% mortality rate, while its progression and persistence lead to sepsis, SIRS, and multiple organ dysfunction syndrome (MODS), increasing its mortality [22,23,24].

Severe acute pancreatitis develops in 20% of cases and leads to SIRS and MODS, with a mortality rate of 8–39% [38]. This sequence of events seems to explain the findings in this experimental model because, based on the present evidence, we can suggest that *Kh* fruit ingestion initially causes severe acute pancreatitis that progresses to SIRS and subsequently to MODS, in which other organs such as the kidney are damaged.

Indeed, the results of our semiquantitative assessment of kidney injury were much lower than those obtained when analyzing pancreatic injury due to *Kh* (Table 2 and Figure 4), and these support our hypothesis.

This report could also be useful in clinical practice when evaluating patients with a history of accidental ingestion of *Kh* fruit, because severe acute pancreatitis could be developing that could evolve into SIRS and subsequently into MODS with fatal consequences. This makes sense and is consistent with reports of fatal cases resulting from cardiorespiratory arrest and respiratory failure caused by the accidental ingestion of a large amount of *Kh* fruit; while patients who consumed less or avoided the seeds developed milder symptoms or neurological symptoms that evolved favorably [3,6,39].

In these cases, efforts could focus on treating the possible development of acute pancreatitis, in order to prevent the development of SIRS and MODS, avoiding possible fatal outcomes in patients with a history of *Kh* ingestion. However, it should be remembered that in this experimental model, an increase in serum amylase was not observed [10], so it is possible that this indicator does not increase in patients either, making diagnosis difficult.

In the future, semiquantitative analyses should be performed in other organs such as the lung and liver in this model of severe acute pancreatitis caused by *Kh* fruit in order to gather more evidence to support our observations.

We could also test other doses of *Kh* fruit to validate our observations in the pancreas, duodenum, and kidney; because there are reports of hyperchromatic nuclei in glomerular cells, edema, and hydropic degeneration in the proximal and distal tubules induced by a dose of 1.25 g/kg of *Kh* [40]; however, the pancreas and duodenum were not analyzed in these studies.

If the behavior observed in the kidney in this work is repeated in other organs such as the liver and lung, in which a lower degree of damage is obtained compared to the damage that occurred in the pancreas, we would be facing a possible selective toxicity of *Kh* on the exocrine tissue of the pancreas and we would justify testing the isolated toxins on a model of pancreatic cancer cells as it was tested on other cell lines [13,14], hoping to obtain a favorable response.

Currently, surgical resection is the only potentially curative treatment for pancreatic cancer, but only 15–20% are technically suitable for radical resection at the time of diagnosis [41]. For this reason, we justify the search for alternative treatments for this neoplasia and we consider that the results of the kidney evaluation in this model of acute pancreatitis induced by *Kh* fruit bring us closer to justifiably testing *Kh* fruit or isolated toxins in models of cancer cells of pancreatic origin in vivo or in vitro.

## 4. Materials and Methods

### 4.1. Kh Fruit Preparation

Ripe *Kh* fruit was collected in Hidalgo, Nuevo León, in July and August. The fruit was air-dried and protected from light, ground, and sieved through a No. 50 mesh sieve (U.S. Standard Sieve Series, Dual Manufacturing Co., Franklin Park, IL, USA). An aqueous suspension was prepared from the ground fruit of *Kh* for administration to the animals in accordance with previous reports [8,9].

### 4.2. Animals and Kh Doses

Animals were obtained from the biotherium of the Monterrey Institute of Technology and Higher Education (ITESM). Eighteen female Wistar rats weighing 250 ± 25 g were divided into six groups (n = 3 each). It was decided to work with a minimum number of 3 units per group to cause the least possible suffering. Five experimental groups were intoxicated with 5 g/kg of body weight of *Kh* fruit in 5 mL of drinking water through an esophageal tube. A control group (n = 3) received only drinking water.

The animals were kept in groups of 3 animals per cage in a bioterium with a 12 h light–dark cycle, with free access to food (LabDiet #5P14-Prolab RMH 2500) and water. The experimental groups were sacrificed at 24, 48, 72, 96, and 120 h after *Kh* administration, respectively. The rats in the control group were sacrificed at 120 h.

All experiments were carried out in accordance with the Mexican Official Standard NOM-062-ZOO-1999 [42] and the Guide for the Care and Use of Laboratory Animals [43]. The protocol was approved by the Research Ethics Committee of the Faculty of Medicine and University Hospital of the Autonomous University of Nuevo León (registration number HT14-004). The only exclusion criterion in this work was the death of the animal before its scheduled sacrifice; no animal was excluded.

### 4.3. Sacrifice and Tissue Preparation

All animals were fasted for 12 h before sacrifice. The animals were sacrificed under anesthesia with intramuscular xylazine (10 mg/kg) followed by an intraperitoneal injection of sodium pentobarbital (88 mg/kg). Kidneys were removed and briefly washed with phosphate buffer, dissected coronally, and immediately immersion-fixed in 4% formaldehyde (pH 7.3). Tissue samples were processed using graded concentrations of isopropylethanol (CTR-01244) and paraffin (Histoplast IM Thermo-Scientific No. 8331, Whaltham, MA, USA) using an Excelsior™ AS tissue processor (Thermo-Scientific, Kalamazoo, MI, USA) and embedded in paraffin blocks using a HistoStar™ embedding processor (Thermo-Scientific, Kalamazoo, MI, USA). Five-μm-thick sections were obtained from each kidney using an HM 355S automatic microtome (Thermo-Scientific, Kalamazoo, MI, USA) and mounted on slides. They were then gradually deparaffinized and rehydrated. The tissue sections were stained with hematoxylin (Richard-Allan Scientific 7212L, Thermo-Scientific, Kalamazoo, MI, USA) and eosin Y (Richard-Allan Scientific 7111L, Thermo-Scientific, Kalamazoo, MI, USA), then dehydrated, clarified, and gradually mounted with Entellan (Merck KGaA, 1079610500, Darmstadt, Germany) for observation and analysis by light microscopy. Additional sections of the same thickness were obtained to perform the TUNEL assay for the detection of fragmented chromatin.

### 4.4. Morphological Analysis

For histological evaluation, 10 random fields from each slide, pre-stained with HE, were analyzed at 40× using a Carl Zeiss light microscope with an integrated HD camera (Carl Zeiss Microscopy GmbH, Göttingen, Germany). The severity of renal damage was assessed using a scoring system for the following parameters: (1) loss of microvilli in proximal tubules, (2) red blood cell extravasation, (3) renal tubular dilatation, (4) tubular necrosis, and (5) presence of casts, as shown in Table 1. Scores of 0–4 were assigned for each field (0 for no lesion, 1 if the lesion occupied <5% of the field, 2 if it was 5–25% of the field, 3 if it was 25–75% of the field, and 4 if it was >75% of the field), according to previous reports of renal damage [16,17]. Three morphologists examined the slides under triple-blind conditions. The scoring tables from the three examiners were processed in Microsoft Excel 2010 for means and standard deviations. The severity of kidney damage caused by *Kh* was determined by summing all scores obtained for each individual parameter, with the highest expected score being 16 points.

### 4.5. Statistical Analysis

The data were first subjected to a normal distribution comparison with the Kolmogorov–Smirnov test, then analyzed by Student’s *t*-test for independent samples with a normal distribution. For the kidney histological damage score, the differences between groups were calculated by the analysis of variance (one-way ANOVA) and a Tamhane’s post hoc test. All data were processed using IBM SPSS version 27 (SPSS, Inc., Armon, NY, USA). Significance was assigned to *p*-values < 0.05 for the kidney histological damage score and for the comparison between renal and pancreatic scores.

### 4.6. Comparison of Kidney and Pancreas Scores

The means and standard deviations of each score obtained in the kidney analysis were converted to percentages. Similarly, the means and standard deviations from the previously reported pancreatic damage [9] were converted to percentages (Table 2). These data were converted considering that the maximum expected score for kidney was 16 points (100%) and the maximum expected score for pancreas was 20 points (100%).

### 4.7. TUNEL Assay

The TACS 2 TdT-DAB in situ apoptosis detection kit (Trevigen 4810-30-K, Minneapolis, MN, USA) was used according to the manufacturer’s instructions. Histological sections, 5 μm thick, were deparaffinized and then rehydrated in PBS at pH 7.4 for 10 min. They were subsequently incubated with proteinase K for 15 min to permeabilize the tissues and washed with ultrapurified water.

Endogenous peroxidase was neutralized by immersing the slides in bleaching solution for 5 min and washing them with PBS for 1 min. A kidney sample from the untreated group was used as an internal control and was incubated with 50 μL of the TACS nuclease enzyme in a humid chamber for 30 min at room temperature and subsequently washed with PBS. All samples, including the positive control, were immersed in the TdT labeling buffer for 5 min and subsequently incubated in a humid chamber with the labeling reaction mixture for 60 min at 37 °C.

Slides were immersed in the Stopping buffer for 5 min to stop the reaction. Histological sections were washed with ultrapurified water and then incubated at 37 °C in a humidified chamber with 50 μL of streptavidin-horseradish peroxidase (Strep-HRP) solution for 10 min. They were then washed with PBS and immersed in diaminobenzidine (DAB) solution for 5 min. Finally, they were washed with ultrapurified water and counterstained with 1% methyl green for 30 s. Once washed, the slides were mounted and analyzed under a brightfield optical microscope.

## 5. Conclusions

In the *Kh*-induced acute pancreatitis model, renal injury is consistent with previous reports and consists primarily of hydropic degeneration and loss of microvilli of the tubular epithelium. However, this model presents a less intense renal histopathological injury than the pancreatic injury. In acute kidney injury, there is no evidence of apoptotic bodies or nuclear chromatin fragmentation in renal glomeruli and tubules in the first 120 h after *Kh* fruit administration. This suggests that *Kh* fruit and/or its toxins could have a potential selective toxicity on pancreatic exocrine tissue, and may be useful in the treatment of pancreatic cancer.

## Figures and Tables

**Table 1:** Kidney injury score per group (mean and standard deviation). The averages of each measurement per parameter are shown in the table. Values marked with * are those with a statistically significant difference compared to the untreated control group (*p* < 0.03).

Parameters Evaluated	Control	24 h ^1^	48 h ^1^	72 h ^1^	96 h ^1^	120 h ^1^
Loss of microvilli	1.16 ± 0.13	1.23 ± 0.17	1.48 ± 0.19 *	1.62 ± 0.28 *	1.64 ± 0.31 *	1.83 ± 0.32 *
Red blood cell extravasation	0.00 ± 0.00	0.00 ± 0.00	0.01 ± 0.03	0.00 ± 0.00	0.00 ± 0.00	0.03 ± 0.05
Tubular dilatation	0.00 ± 0.00	0.00 ± 0.00	0.00 ± 0.00	0.00 ± 0.00	0.13 ± 0.24	0.00 ± 0.00
Necrosis	0.17 ± 0.12	0.30 ± 0.13	0.24 ± 0.19	0.40 ± 0.19 *	0.47 ± 0.10 *	0.51 ± 0.21 *
Cast formation	0.00 ± 0.00	0.01 ± 0.03	0.03 ± 0.07	0.02 ± 0.04	0.00 ± 0.00	0.14 ± 0.16 *
TOTAL SCORE	1.32 ± 0.15	1.54 ± 0.26	1.77 ± 0.22 *	2.04 ± 0.39 *	2.24 ± 0.29 *	2.52 ± 0.60 *

^1^ After treatment with *Kh* fruit.

**Table 2:** Percentage values of the renal injury and pancreatic injury scores in each of the experimental groups and the control group. The significant difference between the percentage values of kidney and pancreatic damage for each group was tested by *t*-Student test. Values marked with * are those with a statistically significant difference (*p* < 0.03).

Scores	Control	24 h ^1^	48 h ^1^	72 h ^1^	96 h ^1^	120 h ^1^
Kidney injury score	1.32 ± 0.15	1.54 ± 0.26	1.77 ± 0.22	2.04 ± 0.39	2.24 ± 0.29	2.52 ± 0.60
Percentage of kidney injury	8.26 ± 0.93%	9.65 ± 1.60%	11.04 ± 1.36%	12.78 ± 2.46%	14.03 ± 1.83%	15.76 ± 3.73%
Pancreas injury score ^a^	0.06 ± 0.17 ^a^	2.96 ± 1.53 ^a^	7.13 ± 2.42 ^a^	13.43 ± 1.05 ^a^	17.06 ± 2.63 ^a^	17.43 ± 2.23 ^a^
Percentage of pancreas injury	0.28 ± 0.83%	14.81 ± 7.64%	35.63 ± 12.05%	67.13 ± 5.27%	85.28 ± 13.14%	87.13 ± 11.17%
Difference between score percentages	7.98% *	5.16% *	24.59% *	54.35% *	71.25% *	71.37% *

^1^ After treatment with *Kh* fruit. ^a^ Obtained from Carcano et al., 2016 [9].

## Data Availability

The data generated in this work are included in the article; further inquiries can be directed to the corresponding author (jaime.garciajr@uanl.edu.mx).

## Abbreviations

The following abbreviations are used in this manuscript:

2LD_50_	twice median lethal dose
DAB	Diaminobenzidine
et al.	and others
g/kg	grams per kilogram
h	hour
HE	Hematoxylin and Eosin staining
*Kh*	*Karwinskia humboldtiana*
mg/kg	milligrams per kilogram
MODS	Multiple Organ Dysfunction Syndrome
PBS	Phosphate-Buffered Saline
SIRS	Systemic Inflammatory Response Syndrome
Strep-HRP	Streptavidin-Horseradish Peroxidase
TACS nuclease	Is a type of enzyme called a DNA endonuclease
TUNEL	Terminal deoxynucleotidyl transferase dUTP nick-end labeling
